# *Chrysomya putoria*, a Putative Vector of Diarrheal Diseases

**DOI:** 10.1371/journal.pntd.0001895

**Published:** 2012-11-01

**Authors:** Steven W. Lindsay, Thomas C. Lindsay, Jessica Duprez, Martin J. R. Hall, Brenda A. Kwambana, Musa Jawara, Ikumapayi U. Nurudeen, Neneh Sallah, Nigel Wyatt, Umberto D'Alessandro, Margaret Pinder, Martin Antonio

**Affiliations:** 1 School of Biological and Biomedical Sciences, Durham University, Durham City, United Kingdom; 2 London School of Hygiene and Tropical Medicine, London, United Kingdom; 3 Natural History Museum, London, United Kingdom; 4 Medical Research Council Unit, Fajara, The Gambia; 5 Institute of Tropical Medicine, Antwerp, Belgium; University of California, Davis, United States of America

## Abstract

**Background:**

*Chrysomya* spp are common blowflies in Africa, Asia and parts of South America and some species can reproduce in prodigious numbers in pit latrines. Because of their strong association with human feces and their synanthropic nature, we examined whether these flies are likely to be vectors of diarrheal pathogens.

**Methodology/Principal Findings:**

Flies were sampled using exit traps placed over the drop holes of latrines in Gambian villages. Odor-baited fly traps were used to determine the relative attractiveness of different breeding and feeding media. The presence of bacteria on flies was confirmed by culture and bacterial DNA identified using PCR. A median of 7.00 flies/latrine/day (IQR = 0.0–25.25) was collected, of which 95% were *Chrysomya* spp, and of these nearly all were *Chrysomya putoria* (99%). More flies were collected from traps with feces from young children (median = 3.0, IQR = 1.75–10.75) and dogs (median = 1.50, IQR = 0.0–13.25) than from herbivores (median = 0.0, IQR = 0.0–0.0; goat, horse, cow and calf; p<0.001). Flies were strongly attracted to raw meat (median = 44.5, IQR = 26.25–143.00) compared with fish (median = 0.0, IQR = 0.0–19.75, ns), cooked and uncooked rice, and mangoes (median = 0.0, IQR = 0.0–0.0; p<0.001). *Escherichia coli* were cultured from the surface of 21% (15/72 agar plates) of *Chrysomya* spp and 10% of these were enterotoxigenic. Enteroaggregative *E. coli* were identified by PCR in 2% of homogenized *Chrysomya* spp, *Shigella* spp in 1.4% and *Salmonella* spp in 0.6% of samples.

**Conclusions/Significance:**

The large numbers of *C. putoria* that can emerge from pit latrines, the presence of enteric pathogens on flies, and their strong attraction to raw meat and fish suggests these flies may be common vectors of diarrheal diseases in Africa.

## Author Summary

While it is well recognized that the house fly can transmit enteric pathogens, here we show the common African latrine fly, *Chrysomya putoria*, is likely to be an important vector of these pathogens, since an average latrine can produce 100,000 latrine flies each year. Our behavioral studies of flies in The Gambia show that latrine flies are attracted strongly to human feces, raw beef and fish, providing a clear mechanism for faecal pathogens to be transferred from faeces to food. We used PCR techniques to demonstrate that these flies are carrying *Shigella*, *Salmonella* and *E. coli*, all important causes of diarrhea. Moreover our culture work shows that these pathogens are viable. Latrine flies are likely to be important vectors of diarrheal disease, although further research is required to determine what proportion of infections are due to this fly.

## Introduction

Controlling diarrheal deaths is crucial to achieve Millennium Development Goal 4; reducing mortality in children under five years by two thirds between 1990 and 2015 [1]. Diarrhea is the second leading cause of death in this age group and is responsible for killing about 1.5 million children each year [2]. In sub-Saharan Africa, diarrhea is caused by a wide range of pathogens including; diarrheagenic (enterotoxigenic [ETEC], enteropathogenic [EPEC] and enteroaggregrative [EAEC]) *E. coli*, serovars of *Salmonella enterica*, *Shigella* spp., *Campylobacter* spp., *Vibrio* spp. and *Aeromonas* spp. One important route of infection is thought to be the mechanical transmission of diarrheal pathogens by flies [3]–[5]. However, despite the long association between flies and pathogens [4] the evidence incriminating flies as vectors of diarrheal pathogens remains weak simply because few adequately controlled studies have been conducted. Moreover, whilst human feces are a rich source of bacteria it is not guaranteed that flies breeding in feces or feeding on feces will be contaminated with high bacterial loads. For example most *Salmonella* spp are destroyed by passage through the acid midgut of the blowfly larva, *Calliphora vicinia* [6]. Bacteria can also be lost during metamorphosis from larva to pupa inside the puparium. At this stage bacteria in the fore and hindgut of the larva lie inside the puparium, outside of the pupa [7], and consequently the fly may emerge from the puparium sterile [4]. Moreover, the normal gut biota of a fly can eliminate potential pathogens as seen with *Salmonella* spp when it is eliminated in the prepupal stage [4].

Of the large number of historical studies investigating the role of flies in the transmission of enteric pathogens, the most convincing was carried out in Texas in communities with high levels of diarrhea [8]. In that context, DDT-sprayed communities had 60% fewer flies (mainly *Musca domestica*, the house fly), 46% lower incidence of diarrhea and a 49% reduction in deaths from diarrhea and enteritis in children under two years old, than unsprayed communities. When the treatment was crossed over, there was a corresponding decline in fly numbers and diarrhea incidence in sprayed communities.

There have been only two recent intervention trials investigating the role of flies in the transmission of diarrheal diseases. Control of *M. domestica* was evaluated in a cross-over study at two military camps in Israel, where fly traps supplemented with insecticidal spraying were used in one camp and not the other. Where the flies were controlled the incidence of diarrheal diseases dropped by 42% and shigellosis decreased by 85% [9]. A community-randomized trial in Pakistan showed that fly control using insecticide reduced the incidence of childhood diarrhea by 23% during periods of high fly densities, where nearly all flies were *M. domestica* [10]. These findings clearly incriminate *M. domestica* as a major vector of diarrheal diseases. In addition, given the cosmopolitan distribution of this fly and the high numbers of adult flies found around human habitation, research on fly-borne diarrheal diseases is dominated by this species.

Exploring the role of other synanthropic flies that may also act as vectors of diarrheal pathogens is also important. Species of *Chrysomya* may be vectors since they are strongly associated with human feces and food. Like *M. domestica*, *Chrysomya putoria* is found across Africa. It was found to be the major fly species in a variety of latrine types in Botswana and Tanzania [11]. In The Gambia, an average pit latrine produces over 100,000 flies each year [12]. Of these flies, 97.8% were identified as *Chrysomya* spp. and only 1.2% *M. domestica*. Since it is common in African markets to see large numbers of adult *Chrysomya* spp on fresh meat and fish we hypothesised that they may mechanically transfer diarrheal pathogens from human feces to food. We explored this hypothesis using odor-baited traps in The Gambia to determine the attractiveness to flies of a variety of breeding and feeding media common in rural villages and by searching for the presence of diarrheal pathogens on flies collected from pit latrines in rural villages. Understanding the routes of diarrhea-pathogen transmission is critically important since it may suggest new opportunities for the control of these life-threatening diseases.

## Methods

### Study Design

This study combines a number of different research methodologies undertaken in the field at different times. We describe the numbers of flies emerging from latrines, their attractiveness to different baits and examine whether they were contaminated with fecal pathogens. These studies are cross-sectional studies, rather than longitudinal ones.

### Routine Fly Collections

Collections were carried out in villages in the Upper River Region of The Gambia between June 2011 and February 2012. This is an area of open Sudanian savannah with a rainy season from June to October followed by a long dry season. Most people live in small rural villages in houses with mud or cement walls and thatched or metal roofs. Toilets are usually pit latrines, although open defecation also occurs. Adult and immature flies were collected from different experiments, locations and seasons to identify common *Chrysomya* spp.

#### Pit latrine emergence traps

Sampling of flies from pit latrines was done in three rural villages, Kundam Demba (13°20′13.51″N, 14°7′3.43″W), Dampha Kunda (13°19′58.62″N, 14°10′37.59″W) and Sare Alpha (13°21′36.84″N, 13°58′49.46″W), in July and November, during the rainy season, and February during the following dry season. Flies emerging from pit latrines were collected by placing mosquito exit traps [13] over open pit latrine drop holes from 09.00h to 09.00h the following day. These traps consisted of a steel-rod framed cube (40 cm×40 cm×40 cm), covered in cotton mosquito netting. The funneled entrance on the bottom face of the trap had netting flaps (40 cm×10 cm) extending outwards from the base to prevent flies crawling out of the trap if the surface of the pit latrine was uneven. On collection the entrance hole of each trap was plugged to prevent any flies from escaping.

#### Odor-baited traps

Flies attracted to food were collected using odor-baited traps made from transparent polypropylene boxes with snap-top white opaque lids box (Whitefurze, Coventry, UK; 17 cm×17 cm×17 cm), perforated with 10, 1.6 cm diameter holes. 50 g of bait media was placed in a white plastic tub (dimensions 9 cm diameter, 6 cm depth, W K Thomas, UK), covered with a cotton-netting lid, secured by an elastic band and placed inside the trap. Few flies entering the traps exited from the holes since they were attracted more to the light entering the transparent sides of the trap. For routine sampling we used raw fish (*Synodontis batensoda*, a common catfish). To collect flies attracted to pit latrines we sealed the drop hole with a lid and vented odors from the latrine into an odor-baited trap. Odors were funnelled through a 10 cm diameter L-bend pipe located 75 cm from the drop hole. The outlet was set flush with the latrine slab and contacted it at a perpendicular angle.

#### Larval collections

Larval specimens were collected by scooping larvae from the surface of the latrine contents. The dipper was made using a metal ladle attached to a 3 m long bamboo cane. Specimens were preserved in 70% ethanol. 13 latrines were sampled in the wet season and 15 in the dry season.

#### Species identification

Adult fly identification was carried out using two keys [14], [15]. Larvae were identified using a combination of characters from Prins [16], which describes larvae of the sister species *C. chloropyga*, and Thyssen [17], which provides an identification key to carrion breeding Diptera from Brazil, including *C. putoria*, and comparing specimens with scanning electron microscope images of *C. putoria* [18].

### Choice Experiments

Odor-baited fly traps were used to assess the relative attractiveness of different breeding and feeding media commonly found in rural Gambia. These studies were carried out at the Medical Research Council's Field Station at Basse Santa Su (13°18′57.96″N, 4°12′31.90″W). For the first experiment the potential attractants were feces from: (1) two Gambian child aged about two years old, (2) three local dogs, (3) a goat, (4) a horse, (5) a cow and (6) a calf. The same animals and children were used throughout. In the second experiment the potential attractants used were: (1) cooked rice, (2) uncooked rice, (3) cut mango, (4) raw beef, (5) raw fish (*S. batensoda*), and (6) human feces from two year old children served as a positive control.

In each experiment 50 g of each media was randomly allocated to six traps positioned along a straight line at 2 m intervals. Traps were rotated between locations on different trapping occasions using a Latin Square design. For the experiment using only feces, traps were left at each position for about seven hours and the entire procedure repeated on 12 occasions. The experiment using different foods was carried out six times for four hours each time. The experiments were carried out for different periods to adjust for differences in monthly fly numbers; for seven hours when fly numbers were low and for four hours when they were high. This sample size was sufficient to detect a 33% difference in fly numbers between traps, at the 5% level of significance and 80% power based on our pilot work (Epi Info version 7.9.7). This *a priori* power calculation was designed to detect large differences in attractiveness between baits since we wanted to identify the major fly attractants. We considered that differences of 33% or more would be sufficient for this purpose.

### Bacteriologic Studies

#### Sample preparation

We sampled for *Shigella* spp on flies collected in the latrines of children with severe diarrhea (three or more loose or liquid stools each day, together with fluid loss which is life threatening) and for a range of potential diarrheal pathogens in latrines of children without severe diarrhea. Children with severe diarrhea were identified through records kept by the Global Enteric Multicentre study (GEMS) of children admitted at the Gambian Government's health clinic in Basse Santa Su.

The DNA extraction and PCR experiments used 30 latrines from Dampha Kunda, Kulukulel (13°17′48.32″N, 14°8′30.18″W) and Tambasanasang (13°22′24.79″N, 14°9′24.46″W). All latrines were used by children experiencing diarrhea during the collection period. Latrines were covered using mosquito exit traps [13] between 08:00h and 09:00h and collected between 18:00h and 19:00h. Traps were transported back to the laboratory and flies killed by freezing at −20°C for 2 hrs and stored at −70°C prior to being processed. Flies were sorted by sex, pooled in groups of 10 and then homogenized with a hand-held tissue homogenizer in 700 µl of distilled water in 2 ml eppendorf tubes. The homogenate was centrifuged for 6 minutes at 14000×g. 110 µl of DNA was extracted from 400 µl of the homogenate suspension using NucliSENS easyMAG (bioMérieux, UK), following the manufacture's protocol and using onboard lysis.

The bacterial culture experiment used 11 latrines from Basse Nding (13°18′16.86″N, 14°12′20.34″W), Dampha Kunda, Demba Kunda Koto (13°15′38.46″N, 14°16′7.41″W) and Kundam Demba. Latrines were covered using exit traps over open pit latrine drop holes from 09:00h to 09:00h the following day. Traps were transported to the laboratory where flies were removed using a mechanical aspirator and placed in a sterile polypropylene box (Whitefurze, Coventry, UK, dimensions 17 cm×17 cm×17 cm).

Wild flies collected from latrines were exposed to agar plates, as described below, to culture bacteria from the flies. We used two types of controls: (1) plates exposed to laboratory-reared flies and (2) plates exposed to the air, with no flies. For the control flies, *Chrysomya* spp. were collected in odor-baited traps baited with raw fish. After 24 hours, the fish, now covered with eggs, was placed between two layers of 2 cm thick cotton wool soaked in tepid tap water and placed into an opaque box (Whitefurze, Coventry, UK, dimensions 17×17×17 cm) covered with cotton netting and kept in the shade outdoors at a temperature of approximately 32°C. The eggs took 7–12 days to develop into adult flies [19].

Wild flies were used for laboratory analysis within two hours of being collected from the latrines. Both wild and those reared from cultures were placed in a freezer at −20°C for 5–8 minutes until stunned. Two flies were added to each agar plate inside a fume hood using sterile forceps and then covered with a plate lid and left for 30 minutes i.e. sufficient time for the flies to become active. We hypothesized that only wild-caught flies would carry fecal bacteria. A further control was performed by opening agar plates in the fume hood. The forceps used to hold flies was moved over the plate to mimic the movement of placing an actual fly on the agar. This control estimates bacterial contamination in the working environment.

#### PCR amplification of diarrheal pathogens

PCRs for the detection of common bacterial strains associated with diarrhea in The Gambia were performed on Q-Cycler (Quanta biotech Ltd, UK). For *Shigella* spp. detection the primers 172.F (5′- ATAGAAGTCTACCTGGCCT -3′) and 172.R (5′- GGGAGAACCAGTCCGTAA -3′) [20] targeting the invasion plasmid antigen H (*ipaH*) were used. The presence of *Salmonella* spp. was investigated using primers invA.F (5′- CTGGCGGTGGGTTTTGTTGTCTTCTCTATT -3′) and invA.R (5′- AGTTTCTCCCCCTCTTCATGCGTTACCC -3′) targeting the invasion gene invA [21]. For the detection of *E.coli* spp. the primers ChuA.F (5′- GACGAACCAACGGTCAGGAT-3′) and ChuA.R (5′- TGCCGCCAGTACCAAAGACA -3′) targeting the heme transport gene ChuA [22] were used. The specificity of primers was tested against *Klebsiella pneumonia*e, *Citrobacter* spp., *Serratia* spp. and *E. coli*. The master mix for all reactions contained 0.2 µM (PCR1 contained 0.4 µM) of the forward and reverse primers, 3 mM of MgCl_2_, 1.25 U of Taq polymerase (Qiagen, Crawley, UK), 1× Buffer (Qiagen, Crawley, UK), 0.4 mM of dNTPs (Fermentas, Nottingham, UK) and nuclease-free water made up to a total volume of 23 µl to which 2 µl of extracted DNA was added per reaction. For detection of *Shigella* spp. and *E.coli* spp. the amplification programme was as follows: initial denaturation at 95°C for 10 minutes, followed by 30 cycles of 95°C for 30 seconds, 57°C for 30 seconds and 72°C for 45 seconds and a final extension of 72°C for 10 minutes. For detection of *Salmonella* spp. the amplification programme was as follows: Initial denaturation at 94°C for 10 minutes, followed by 30 cycles of 94°C for 30 seconds, 55°C for 60 seconds and 72°C for 60 seconds and a final extension of 72°C for 10 minutes.

A multiplex PCR was used for differentiation of enterovirulent *E. coli* (see table 1): ETEC using primers targeting *eltB* and *estA* responsible for the production of LT and ST enterotoxins respectively; EPEC using primers targeting the *E. coli* attaching and effacing (*eae*) and bundle-forming pilus (*bfpA*) adhesion genes; and EAEC using primers targeting the plasmid encoded gene *aatA* from the pAA and the chromosomal gene *aaiC*. The master mix containing the reverse and forward primers and 1.25 U of *Taq* polymerase (Qiagen, Crawley, UK); 2 mM of MgCl_2_; 1.25 mM of dNTPs (Fermentas, Nottingham, UK); and nuclease-free water made up to a total volume of 17 µl to which 3 µl of extracted DNA was added per reaction. Thermal cycling was performed in the Gradient Palm–Cycler (Corbett Life Sciences, Cambridge, UK), under the following conditions: Initial denaturation at 96°C for 4 minutes, followed by 35 cycles of 95°C for 20 seconds, 57°C for 20 seconds and 72°C for 1 minute and a final extension of 72°C for 7 minutes. DNA amplified by all PCRs was separated on an agarose gel (1.5–2%) stained with 500 ng/µl ethidium bromide and using Tris-borate-EDTA (TBE) as running buffer at 100 V for 70 minutes. A transilluminator (Gel Doc XR system, Bio-Rad, Hertfordshire,UK) was used for visualisation. In cases where amplification signals were weak, amplicons were re-analysed using the Qiaxcel Advanced System (Qiagen, Crawley, UK).

**Table 1:** Primer sequences and the expected amplicon sizes for the PCR for detection of diarrheagenic *E. coli* [45].

Strain of *E. coli*	Target Gene	Location	PCR size (bp)	Primer sequence (5′-3′)
ETEC	ST	Plasmid	147	GCTAAACCAGTAGAG(C)TCTTCAAAA- -CCCGGTACAG(A)GCAGGATTACAACA
ETEC	LT	Plasmid	508	GCACACGGAGCTCCTCAGTC TCCTTCATCCTTTCAATGGCTTT
EPEC	*eae*	Chromosome	881	CTGAACGGCGATTACGCGAA CGAGACGATACGATCCAG
EPEC	*bfpA*	Plasmid	300	AATGGTGCTTGCGCTTGCTGC GCCGCTTTATCCAACCTGGTA
EAEC	*aatA*	Plasmid	650	CTGGCGAAAGACTGTATCAT CAATGTATAGAAATCCGCTGTT
EAEC	*aaiC*	Chromosome	215	CTTCTGCTCTTAGCAGGGAGTTTG AAGCGTGAAATGCCTGAGGA

#### Bacterial culture

We used three different solid culture media: Xylose-Lysine-Deoxycholatye (XLD) Oxoid (Basingstoke, UK), MacConkey (MAC) (Sigma-Aldrich, Dorset, UK) and Campylobacter (CAMPY) Oxoid (Basingstoke, UK). After the inoculation of the culture media by the flies, the MAC and XLD media were streaked and incubated. All plates were incubated at 37°C overnight except the Campy plates that were incubated at 42°C together with a Campy gas pack for 48 hours. From the primary MacConkey agar plate, three suspected morphologically different colonies of *E. coli* were purified and incubated overnight. The growth of purified colonies were subjected to API 20E to identify for *E. coli* by selecting 3–4 colonies for (2 McFarland standard) suspension.

Flies were examined for the presence of bacterial pathogens, including *Shigella* spp., *Salmonella* spp, *Campylobacter* and diarrheagenic *E. coli* (subtypes EAEC, EPEC and ETEC). All cultured agar plates, excluding the CAMPY plates, were examined after 24 h for growth and identification of enteric pathogens. CAMPY plates were examined after 48 h for *Campylobacter* growth and identification. Lactose and non-lactose fermenting isolates on MAC plates were subjected to biochemical identification test using Analytical Profile Index (API) 20E (bioMerieux cat#20160). Suspected isolates for *Campylobacter* spp. were tested against oxidase, catalase and gram reactions. Confirmed *E. coli* isolates were harvested and stored at −70°C to later be differentiated by strain.

### Statistical Analyses

General linear modelling was used to account for the variation in fly numbers due to trap bait, position of trap and replicate using SPSS version 19.0. Comparisons between different baits were made using Bonferonni corrections to account for multiple comparisons. Comparisons between proportions were made using the Chi-square test in Epi Info version 7.9.7. Population prevalence rates of infection were estimated from pooled samples based on the bias-corrected maximum likelihood estimation, using a skewness-corrected score for estimating 95% confidence intervals. An excel add-in available from the West Nile Virus website of the Centers for Disease Control and Prevention was used to compute these values [23].

### Ethical Procedures

Written informed consent was provided by householders for fly collections from latrines and by the parents and guardians of children for donation of stool samples. Ethical approval for this study was provided by the Gambian Government/Medical Research Council's Laboratories Joint Ethics Committee in The Gambia (SCC 1234v and 21238v2) and the London School of Hygiene and Tropical Medicine's Ethics Committee (010/243 and 5984). An animal protocol was not necessary since no animals were handled during the study. Animal faeces were collected from the ground.

## Results

### Routine Fly Collections

**Figure 1:**
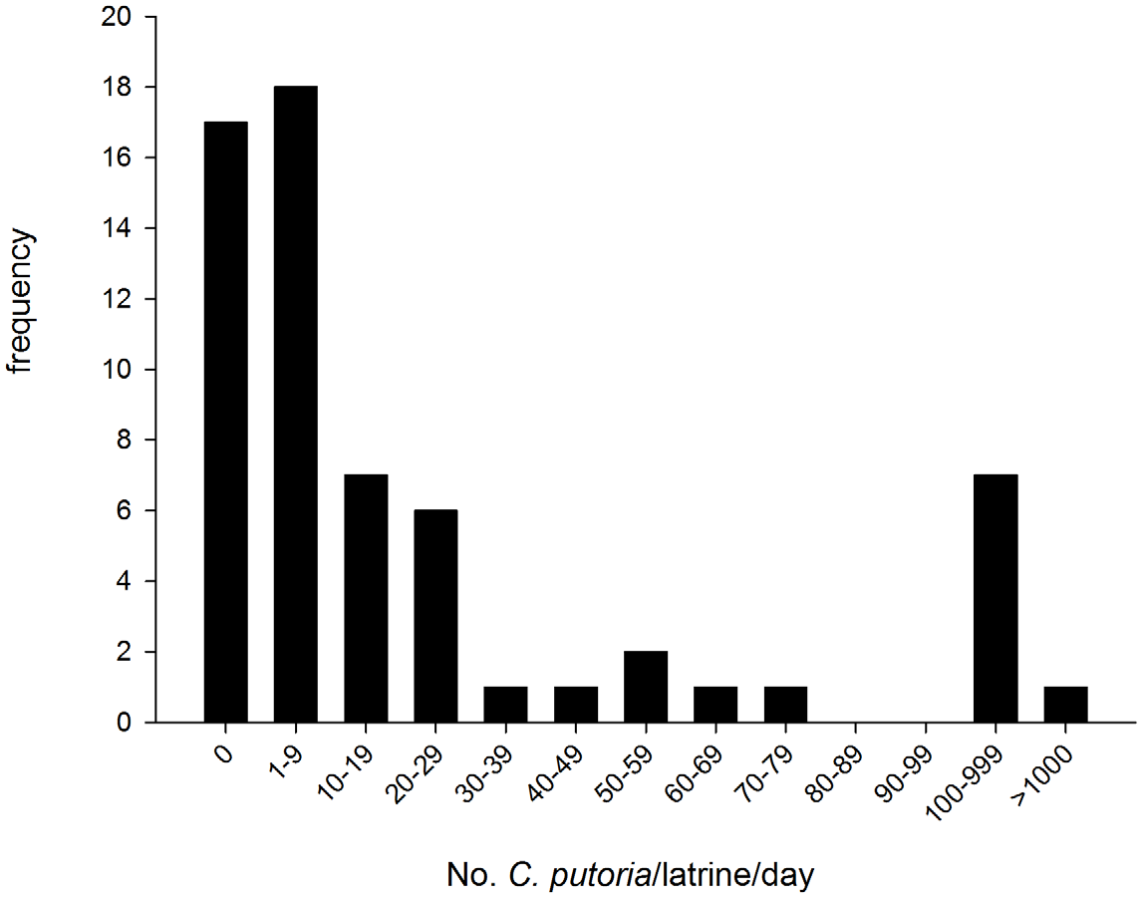
Frequency of flies collected from 62 pit latrines in the rainy season.

A total of 4,572 flies were collected from 62 pit latrines during July and November (Figure 1; median = 7.00 flies/latrine/day, interquartile range, IQR = 0.0–25.25). We found 13% (n = 8) of latrines sampled produced 85% of the total flies (n = 3689). 60% (n = 37) produced less than 10 flies. Of the 4,034 flies collected from 31 latrines in July, 94.72% were *C. putoria*, 5.21% *Musca* spp., 0.05% *C. marginalis* and 0.02% *Sacrophaga* spp. Of the two morphologically similar species of adult *Chrysomya*, *C. putoria* was the dominant species in both the wet season (99.2%, 891/898; Table 2) and dry season (99.1%, 1173/1183). Since nearly all flies collected were *C. putoria* we refer, for simplicity, only to this species hereafter. Overall 80.9% of the *C. putoria* were females, with a greater proportion of females emerging from latrines in the dry season (91.5%) than wet season (74.5%, χ^2^ = 6.017, p = 0.0142) and captured in fish-baited traps in the wet season (91.7%) compared with the dry season (77.1%, χ^2^ = 12.44, p<0.001).

**Table 2:** Collection of *Chrysomya* spp using different sampling methods.

Sampling method	Wet season					Dry season				
	n	*C. putoria*		*C. albiceps*		n	*C. putoria*		*C. albiceps*	
		Females	Males	Females	Males		Females	Males	Females	Males
Adult flies										
Latrine exit traps	32	531 (74.5%)	182^a^ (25.5%)	5 (71.4%)	2 (28.6%)	3	43 (91.5%)	4^a^ (8.5%)	0 (0%)	0 (0%)
Odor-baited latrine traps	5	50 (87.7%)	7 (12.3%)	0 (0%)	0 (0%)	72	467 (89.8%)	53 (10.2%)	0 (0%)	0 (0%)
Fish-baited traps	12	111 (91.7%)	10^b^ (8.3%)	0 (0%)	0 (0%)	72	467 (77.1%)	139^b^ (22.1%)	10 (100%)	0 (100%)
Total	48	692 (77.7%)	199 (22.3%)	5 (71.4%)	2 (28.6%)	147	977 (83.2%)	196 (16.8%)	10 (100%)	0 (0%)
Larvae										
Latrine dipping	13	357 (100%)	0 (0%)	15	289 (100%)	0 (0%)				

Letters ^a^ and ^b^ denote significant differences between the sex ratios of flies collected in different seasons, where a corresponds to p = 0.0142 and b with p<0.001; n = number of sampling occasions. Note that flies sampled from latrines in July are not included in the table since they were not separated into *C. putoria* and *C. albiceps*. For each sampling method surveys were each made over one to three weeks in the wet and dry season.

The larvae of *Chrysomya* spp collected in pit latrines were all *C. putoria*. None of the samples included larvae of *C. albiceps*, which are highly distinctive due to their fleshy processes [18]. The percentage of Psychodidae compared to *C. putoria* was 7.75% (30/387) in the wet season and 61.47% (461/750) in the dry season. These flies were found mainly in different latrines from *C. putoria*, preferring latrines with relatively clear water compared to the more solid contents.

### Choice Experiments

**Figure 2:**
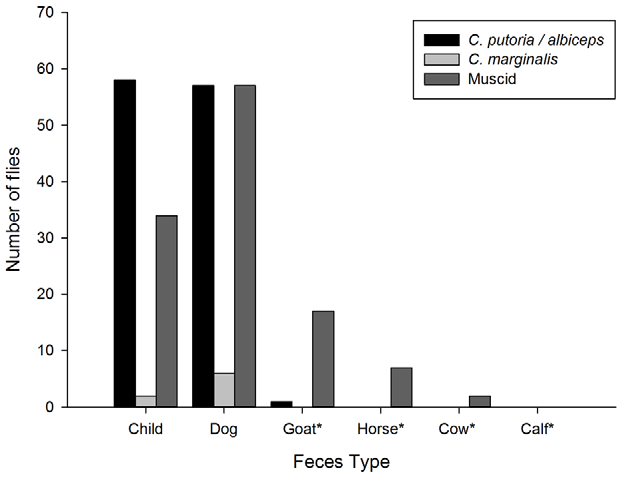
Relative attractiveness of different feces to flies. Where *Indicates a significant difference between the attractiveness of human feces and other media.

**Figure 3:**
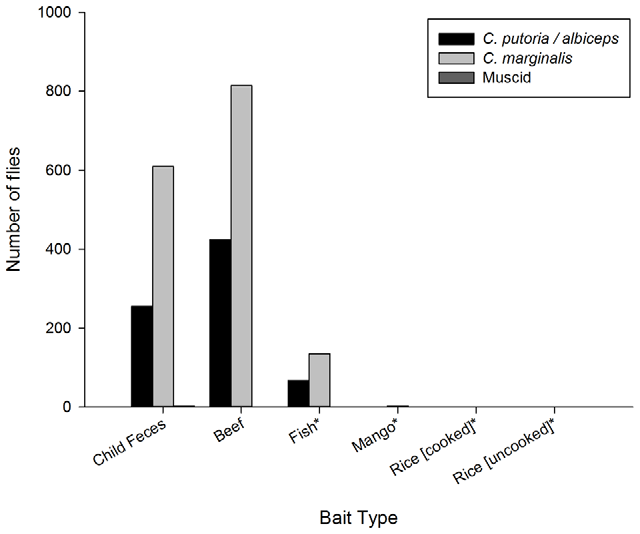
Relative attractiveness of different foods and child feces to flies. Where *Indicates a significant difference between the attractiveness of human feces and other media.

The number of *C. putoria* collected from traps baited with the feces of different species varied significantly (Figure 2, F = 18.04, p<0.001). There was a significant difference between human feces and goat, horse, cow and calf feces (p<0.001). More flies were collected from traps with feces from young children (median = 2.5, IQR = 1.0–8.5) and dogs (median = 1.0, IQR = 0.0–12.0) than from herbivores (median = 0.0, IQR = 0.0–0.0; goat, horse, cow and calf; p<0.001). There were significant differences in the number of *C. putoria* collected from different foods and human feces (Figure 3, F = 30.01 p<0.001). Most were collected in traps baited with raw beef, human feces and raw fish. There was no difference between human feces and beef (z = −0.943, P = 0.345). Flies were strongly attracted to raw meat (median = 44.5, IQR = 26.2–143.0) compared with fish, cooked and uncooked rice, and mangoes (median = 0.0, IQR = 0.0–0.0; p<0.001).

### Bacteriologic Studies

#### PCR amplification of diarrheal pathogens

5,010 *Chrysomya* spp collected from Dampha Kunda, Kulukuleh and Tambasanasang were homogenized in batches of ten. Nucleic acids were purified and used for the PCR detection of diarrheal pathogens. *Escherichia coli*, *Salmonella* spp and *Shigella* spp were detected in 27%, 1.4% and 0.6% of samples respectively; with 7.5% of the *E. coli* detected being EAEC strains, whereas no ETEC or EPEC were detected by PCR. This corresponds to population prevalence rates of 30.3% (95% Confidence intervals = 25.5–35.6%) for *E. coli*, of 1.4% (95% CIs = 0.61–2.72%) for *Salmonella* spp. and 0.59% (0.15–1.59%) for *Shigella* spp.; with 7.64% (95% CIs = 3.9–7.6%) of *E. coli* being EAEC strains.

#### Bacterial culture

Of the 72 MAC and XLD plates exposed to flies from latrines, 21% grew *E. coli* (n = 15), 35% *Pseudomonas* spp (n = 25) and 38% *Proteus* spp (n = 27). There were two ETEC pathogens, one expressing the heat-labile toxin (LT) and the other expressing both the LT and heat-stable toxin (ST). Of the 34 CAMPY plates exposed to flies from latrines, 38% grew *Bacillus* species (n = 13). Of the three plates exposed to artificially-reared flies, two grew *Proteus* spp and one *Bacillus* spp. There was no growth on any of the 18 control plates (MAC = 6, CAMPY = 6, XLD = 6).

## Discussion

Our findings support the hypothesis that *C. putoria* is a putative vector of diarrheal diseases since 21% of this species emerging from latrines carried fecal bacteria, including pathogens that cause diarrhea. Moreover, since these flies are also strongly attracted to raw meat and fish, they are likely to contaminate foodstuffs with bacterial pathogens. Although meat and fish are not eaten raw in The Gambia, we speculate that pathogens could be transferred to the mouth and other foodstuffs after handling contaminated meat.

*Chrysomya putoria* is known as the tropical African latrine blowfly [19], and was the dominant fly species emerging from pit latrines in our study. They are common in sub-Saharan Africa [24], with recent incursions into South America [25]. *Chrysomya putoria* have been reported as the dominant fly from pit latrines in Dar es Salaam, Tanzania [26], Kinshasa, Democratic Republic of Congo [27], Sudan [28] and Zimbabwe [29]. The first, and only, previous record of *C. putoria* in The Gambia was from one ‘modern’ lavatory in Sukuta, on the coast, in July 1952 [30]. The adults of *C. putoria* are morphologically similar to *C. albiceps*, and we think that recent descriptions of *C. albiceps* emerging from pit latrines in The Gambia [12] are likely to be mistaken; meaning *C. putoria* is the dominant fly in the country, not *C. albiceps*. *Chrysomya putoria* are known to breed in wet feces and can liquefy large fecal masses [31], which help break down feces and increase the longevity of latrines. We found 13% of latrines produced 85% of the flies, which is roughly comparable with Pareto's principle, that 80% of disease transmission results from 20% of hosts, or in this case, latrines [32]. It may be that the large number of latrines that did not produce flies were too dry although this requires further investigation that may lead to identifying potential fly interventions.

**Figure 4:**
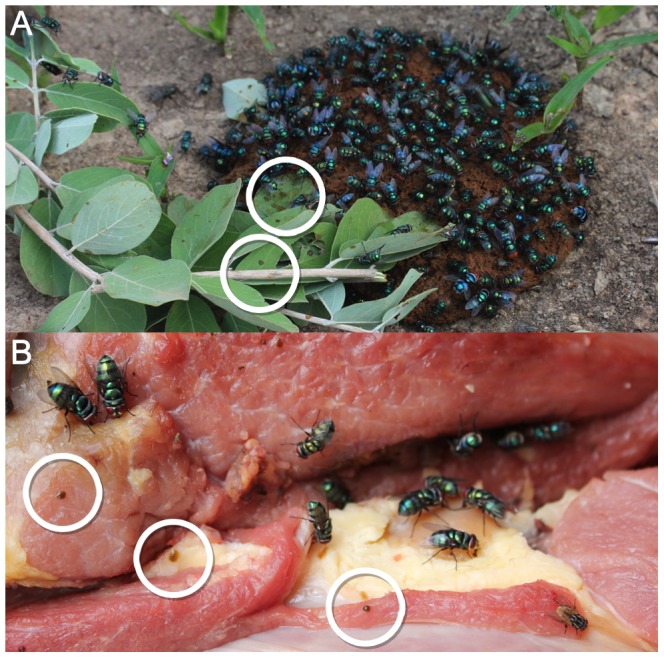
*Chrysomya putoria* dominating the surface of A human feces and B uncooked market stall meat. The rings demonstrate vomit drops and fly feces.

Our choice of experiments demonstrates that *C. putoria* are attracted strongly to both human and dog feces. This attractiveness is graphically illustrated when finding human feces deposited in the open; these, and the nearby vegetation, are usually covered rapidly by *C. putoria*, (Figure 4). We also demonstrated that *C. putoria* is attracted to raw beef. Although our experiments did not find that flies were attracted strongly to fish, this was probably because in this choice experiment flies preferred feces and raw meat. We know from other work that fish can be used as bait for *C. putoria*. Both meat and fish are common and major sources of protein in rural Gambia. Our findings support the general view that *C. putoria* is a blowfly attracted to feces and carrion [24], and these sources of protein are essential for egg development [19].

We demonstrated by PCR that pools of *Chrysomya* collected emerging from pit latrines were contaminated with human fecal bacteria, including the important human pathogens: *E. coli* (27%), EAEC (2%), *Salmonella* spp (1.4%) and *Shigella* spp (0.6%). We also showed that these bacteria are likely to be viable since we were able to culture *E. coli* directly from 21% of plates where live flies had been introduced. These levels of infection are comparable to the 0.5% *Chlamydia trachomatis* infection rate found for *Musca sorbens* [33], which is responsible for 56% of trachoma cases in Gambian children [34]. We know of only one study reporting enteric pathogens (polio, coxsackievirus, *E. coli* and *Salmonella* spp.) from *C. putoria* and this was from Madagascar over 50 years ago [35].

There are limitations to our study design. We cannot exclude the possibility that cross-contamination occurred between flies collected in the same trap or from the trap itself thereby inflating the true bacterial carriage prevalence rate. Moreover, the carriage of pathogens does not prove conclusively that *Chrysomya* spp are mechanical vectors of diarrheal pathogens. This evidence must come from a randomised-controlled trial. Nonetheless, we demonstrated that only culture plates with flies collected from latrines were contaminated with fecal bacteria, not those exposed to flies grown without feces, nor those plates which were manipulated in a way to simulate fly introduction.

Although we suggest that for *C. putoria* the mechanism of transmission is indirect, via the contamination of foodstuffs, rather than direct transmission, as is the case for trachoma, the infection rates found in our study suggest that these flies are likely to be common mechanical vectors of diarrheal pathogens in The Gambia and other parts of sub-Saharan Africa. Moreover, they can be found emerging in prodigious numbers from latrines at a rate of 74/flies/latrine/day reported in our pilot study and 274/flies/latrine/day reported by Emerson and colleagues during a year-long survey of latrines in The Gambia [12]. If each latrine produces 100,000 flies each year [12], with most compounds containing at least one latrine, this represents an enormous capacity to transfer fecal pathogens to food. Calliphorids, like *C. putoria*, are generally long lived with laboratory colonies surviving an average of three to four weeks [36] and many may return to feed and lay eggs on feces during their life time increasing their opportunity to be contaminated with diarrheal pathogens. Coupled with *E. coli*'s persistence outside a host of more than one month [37], these flies have the potential to be vectors for substantial periods. This is further enhanced by the long distance dispersal of these flies normally ranging from 0–6 km, with a maximum dispersal of >16 km in 12 days [38]. Our findings suggest that around 1,000 infected flies will land on an average piece of meat (assuming regular replacement) over one year, assuming a 2% carriage of enteric pathogens, with a median of 44.5 flies landing on 50 g of raw meat in 4 hours, assuming fly contact was at a similar rate for 12 h each day (i.e. [44.5×3×365]×0.02). Whilst we acknowledge that cooking will kill bacteria on meat and fish, we suggest that those handling contaminated food pass the bacteria on to others on their hands and common household items. Dirty hands are a well-known route of transmission and it has been shown that hand-washing can reduce the risk of diarrheal incidence by 48% [39].

**Figure 5:**
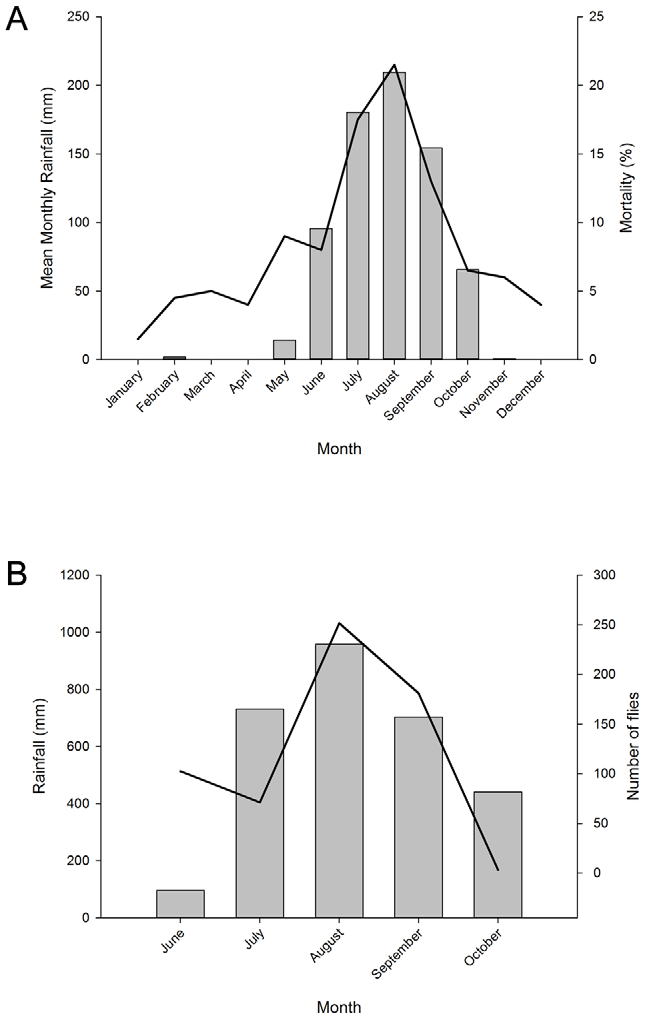
Seasonal relationship between rainfall, childhood deaths from acute gasteroenteritis and latrine flies. A. Seasonality of deaths in children under 5 years old due to acute gastroenteritis and mean monthly rainfall in the Upper River Region, The Gambia between 1989–1993. Child mortality data were taken from [40] and rainfall data for the study area were from the Gambian Governments meteorological station at Basse Santa Su. B. Seasonality of four village fly populations and monthly rainfall in the Farafenni area in 1997, The Gambia. Fly population data were taken from [12] and rainfall data were from the MRC Field station at Farafenni.

How important might *C. putoria* be as a vector contributing to diarrheal diseases mortality? An examination of the seasonality of deaths from acute gastroenteritis [40] and the seasonality of numbers of *C. albiceps* (we suggest is *C. putoria*) collected from latrines in different parts of The Gambia in different years (Figure 5) shows that both fly numbers and diarrheal deaths rise and fall at the same time of year. There are two major qualifications with this analysis. Firstly, association does not prove causality, and there may be other reasons for the seasonality in diarrhea deaths. Secondly, the data may be biased since they were drawn from studies conducted in different places at different times. Nonetheless the shared seasonality of flies and diarrheal deaths, merits further investigation.

Crucially, if *C. putoria* are important vectors, their control may be relatively simple and targeted at the source of the problem; the pit latrine. Targeted control of the small number of most prolific latrines will dramatically reduce major breeding sites. The construction of Ventilated Improved Pit latrines (VIPs) [41], the use of insecticides [42] or using the odors of the latrine as a natural bait (unpublished data) may lead to dramatic reductions in fly numbers. Nonetheless this will only be effective if combined with health programmes that reduce open defecation. At present 5% of the Gambia's rural population still practice open defecation [43]. Moreover, in recent focus group discussions with village latrine users in Kundam Demba (unpublished data), it was revealed that children under five years old were not allowed to use their latrines. Mothers forbade their children using the latrine, fearing they would make it dirty “especially when they have diarrhea”. Another study in The Gambia suggested that it is the last-born child that is prevented from using the latrines, rather than the actual age of the child [44]. Young children were expected to openly defecate and the mothers to dispose of the feces in their latrines. This left a period between defecation and disposal when the feces would be exposed to the open air and flies. Open defecation is likely to increase feeding and breeding opportunities for *Chrysomya* spp therefore increasing their potential as diarrheal vectors. Any control programmes to reduce fly numbers should also tackle open defecation.

Here we suggest that *C. putoria* may be of major public health importance as mechanical vectors of diarrheal pathogens. Since this fly is widely distributed across sub-Saharan Africa and parts of South America this hypothesis should be of wider importance since diarrheal diseases are a major cause of childhood morbidity and mortality in these regions. Whilst transmission of diarrheal diseases by flies is typically associated with the house fly, *M. domestica*, here we suggest that the strong association between the blowfly *C. putoria* and human feces, combined with its strong attraction to raw meats, may make it a putative mechanical vector of enterovirulent pathogens. Intervention trials are needed to establish the role of *C. putoria* as a mechanical vector of diarrheal pathogens.
